# The prevalence of influenza bacterial co-infection and its role in disease severity: A systematic review and meta-analysis

**DOI:** 10.7189/jogh.13.04063

**Published:** 2023-06-16

**Authors:** Mengling Qiao, Gary Moyes, Fuyu Zhu, You Li, Xin Wang

**Affiliations:** 1 School of Public Health, Nanjing Medical University, Nanjing, Jiangsu, China; 2 Usher Institute, The University of Edinburgh, Edinburgh, United Kingdom

## Abstract

**Background:**

Evidence suggests that influenza bacterial co-infection is associated with severe diseases, but this association has not been systematically assessed. We aimed to assess the prevalence of influenza bacterial co-infection and its role in disease severity.

**Methods:**

We searched PubMed and Web of Science for studies published between 1 January 2010 and 31 December 2021. We performed a generalised linear mixed effects model to estimate the prevalence of bacterial co-infection in influenza patients, and the odds ratios (OR) of death, intensive care unit (ICU) admission, and requirement of mechanical ventilation (MV) for influenza bacterial co-infection compared to influenza single-infection. Using the estimates of OR and prevalence, we estimated the proportion of influenza deaths attributable to bacterial co-infection.

**Results:**

We included 63 articles. The pooled prevalence of influenza bacterial co-infection was 20.3% (95% confidence interval (CI) = 16.0-25.4). Compared with influenza single-infection, bacterial co-infection increased the risk of death (OR = 2.55; 95% CI = 1.88-3.44), ICU admission (OR = 1.87; 95% CI = 1.04-3.38), and requirement for MV (OR = 1.78; 95% CI = 1.26-2.51). In the sensitivity analyses, we found broadly similar estimates between age groups, time periods, and health care settings. Likewise, while including studies with a low risk in confounding adjustment, the OR of death was 2.08 (95% CI = 1.44-3.00) for influenza bacterial co-infection. Based on these estimates, we found that approximately 23.8% (95% uncertainty range = 14.5-35.2) of influenza deaths were attributable to bacterial co-infection.

**Conclusions:**

We found that bacterial co-infection is associated with a higher risk of severe illnesses compared to influenza single-infection. Approximately one in four influenza deaths could be attributable to bacterial co-infection. These results should inform prevention, identification, and treatment for suspected bacterial co-infection in influenza patients.

**Registration:**

PROSPERO CRD42022314436.

The influenza virus is a common respiratory virus which caused approximately 290 000-650 000 deaths between 1999 and 2015 globally [1]. In 2018, the World Health Organization (WHO) reported that there were 3-5 million new episodes of severe influenza illnesses worldwide [2]. Evidence shows that the mechanism underlying the synthetic interaction between influenza and bacteria is complex. For example, influenza virus infection can cause epithelial damage and functional changes in lungs, and increase receptor availability for bacterial adherence, facilitating bacterial invasion and leading to decreased clearance of bacterial infection. It can also interfere with lung immune responses to bacterial invaders, impairing bacterial recognition and clearance. The synergy of influenza and bacteria leads to dysregulation of host immune responses and increased inflammation, causing severe complications [3,4]. Epidemiological data show that 20-60 million of the influenza patients died from bacterial co-infection during the 1918 influenza pandemic [5], and 23% of fatal influenza cases were co-infected with bacteria during 2009 influenza pandemic [6]. Evidence suggests that bacterial co-infection is linked to severe complications, yet no systematic review has synthesised epidemiological data to assess the association between severe complications and influenza bacterial co-infection. We therefore conducted a systemic review and meta-analysis to explore whether influenza bacterial co-infection was associated with more severe outcomes compared to influenza single infection. We focused on studies published in the past decade during which the new influenza subtype virus H1N1pdm09 has displaced the previous seasonal H1N1 subtype and began to circulate and cause seasonal epidemics worldwide. We had two objectives: to estimate the prevalence of influenza bacterial co-infection and to assess the association of severe outcomes with influenza bacterial co-infection compared to influenza single-infection.

## METHODS

### Literature review

We conducted a systematic review in accordance with the Preferred Reporting Items of Systematic Reviews and Meta-Analyses (PRISMA) guideline to estimate the prevalence of bacterial co-infection among patients with laboratory-confirmed influenza infection, and to assess the role of bacterial co-infection in the disease severity (PROSPERO CRD42022314436). We searched Web of Science and PubMed for observational studies published between 1 January 2010 and 31 December 2021 using a combination of influenza-and bacteria-related terms (Table S1 in the **Online Supplementary Document**) without restrictions on languages or countries.

### Eligibility criteria and case definitions

We included studies reporting data on laboratory-confirmed influenza infection and bacterial co-infection. We defined influenza-bacterial co-infection as the identification of bacteria in respiratory tract samples, urine, and sterile sites (e.g. blood) in patients with laboratory-confirmed influenza (Table S2 in the **Online Supplementary Document**). Patients without laboratory-confirmed bacteria were referred to as having influenza single-infection. The included studies must have had any of the following data: the prevalence of bacterial co-infection among patients with influenza infection, the odds ratio (OR) of being admitted to intensive care unit (ICU), the OR of death, the OR of requirement of mechanical ventilation (MV) among people with bacterial co-infection compared to those with influenza single-infection, or the length of hospital stay (LOS) in patients with bacterial co-infection and those with influenza single-infection. Where other risk measures (e.g. risk ratio) were reported, we transformed them to ORs using the raw data in the study. We excluded studies if they had less than 10 influenza cases, only reported data on high-risk population subgroups (e.g. with underlying conditions), had an unclear case definition, or were duplicates of the included studies. Only a few studies reported data in outpatient settings, probably due to testing being less common in outpatient settings, so we excluded them from this systematic review; we only included the studies conducted in emergency departments (ED), general wards, and ICUs. Case definitions commonly used in the studies were hospitalised influenza-like illness, acute respiratory infection, and pneumonia.

Two reviewers (MQ and GM) independently screened the titles and abstracts for eligibility, followed by the full texts for the final inclusion decision. Three reviewers (MQ, GM, FZ) extracted the data for all included studies using a pre-specified extraction form. We resolved discrepancies in all steps through discussion with another reviewer.

We used Microsoft Excel worksheets to extract the following information for each study: article information (e.g. author, publication year, and title), age of participants, study location, study period, study design (retrospective, prospective), health care settings (e.g. ED, general ward, ICU), inclusion and exclusion criteria, case definitions, community- or hospital-acquired infection, type and subtype of influenza viruses, test methods, and specimens for influenza and bacteria. We also extracted the number of patients who had influenza infection or bacterial co-infection for all studies. To compare disease severity between influenza single-infection and bacterial co-infection, we extracted the number of patients with influenza single-infection or with influenza bacterial co-infection, and the number of patients with any severe outcome (i.e. admission to ICU, requiring MV, and mortality) in the two groups. Where available, we extracted the adjusted odds ratio of any of the severe outcomes between patients with bacterial co-infection and those with influenza single-infection. We also extracted the mean estimates and the standard deviation (SD) of the LOS, or the median estimates and the interquartile range (IQR) in the two groups where available.

### Quality assessment

We assessed the studies for potential bias using a modified Newcastle-Ottawa Scale (NOS) (Table S3 in the **Online Supplementary Document**), following previous literature [7,8]. This scale contains five main domains: study design, representativeness of the study population, influenza test methods, testing levels for bacteria, and bacterial confirmation. For studies reporting data on disease severity, we assessed two additional domains: adjustment for confounding and accuracy of the length of hospital stays. We separately conducted critical assessments on the risk of bias (low or high) for each domain.

### Statistical analysis

We conducted meta-analyses to estimate the prevalence of bacterial co-infection in patients with laboratory-confirmed influenza infection and the OR of any of the severe outcomes (i.e. admission to ICU, requiring MV, and mortality) in patients with influenza bacterial co-infection compared to those with influenza single-infection using a generalised linear mixed-effects model [8,9]. For the LOS, we converted median estimates to means [10,11]. We used funnel plots to assess small-study effects. We considered a two-sided *P* < 0.05 was as statistically significant. We conducted all analyses using R software (version 4.2.0).

We conducted subgroup analyses by age of patients (≤18 years old, >18 years old), study period (before, during, and after the 2009 influenza pandemic), study design (retrospective; prospective), health care settings (ED, general wards, or ICU). We performed sensitivity analyses where applicable by restricting to studies with a low risk of bias in the test methods used to detect influenza viruses, restricting to studies with a low risk of bias in the methods for bacterial confirmation, excluding studies with a high risk of bias in the adjustment for confounding, excluding influential studies based on Cook’s distance [12], or excluding small studies with <50 influenza cases.

Using estimates of prevalence and ORs, we estimated the proportion of influenza deaths attributable to bacterial co-infection using Levin’s formula for attributable fraction estimation [13,14]. Following previous studies [8], we estimated the 95% uncertainty range (UR) of the estimates based on 1000 samples from log-normal distributions of meta-estimates of the prevalence and ORs, with the 2.5th percentile as the lower bound and 97.5th percentile as upper bound.

## RESULTS

### Search results

**Figure 1:**
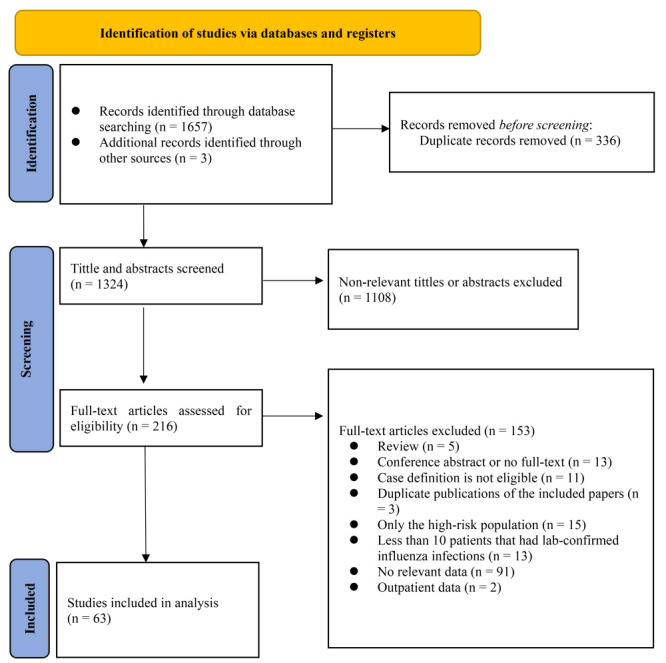
Flow diagram of study selection.

We retrieved 1660 records from the literature search. After deduplication, we screened the titles and abstracts of 1324 records and excluded 1108 for not meeting the eligibility criteria; we assessed the full texts of the remaining 216 studies (**Figure 1**). Sixty-three articles reported eligible data on the prevalence of bacterial co-infection in influenza infection, while 23 reported eligible data on the disease severity of influenza single-infection and bacterial co-infection (**Table 1** and Table S4-S5 in the **Online Supplementary Document**).

**Table 1:** Meta-estimates of the prevalence of influenza bacterial co-infection and OR of death for influenza bacterial co-infection compared to influenza single-infection

	Prevalence of bacterial co-infection			OR of death for bacterial co-infection		
	**n**	**Prevalence, % (95% CI)**	***P*-value**	**n**	**OR (95% CI)**	***P*-value**
**All studies**	63	20.3 (16.0-25.4)		20	2.55 (1.88-3.44)	
**Subgroup analyses**						
**Age group**						
<18 y	15	23.0 (13.8-35.7)	0.99	2	2.41 (0.72-8.00)	-
>18 y	21	22.5 (16.5-30.0)	Ref	9	2.11 (1.74-2.56)	Ref
Unstratified	27	16.5 (12.2-22.0)	0.18	9	3.39 (1.77-6.54)	0.19
**Time period**						
Pre-pandemic	8	11.7 (3.8-30.6)	0.08	1	NA	-
During-pandemic	15	16.3 (9.2-27.2)	0.32	6	1.91 (0.89-4.11)	0.21
Post-pandemic	18	22.5 (15.7-31.3)	Ref	6	2.77 (2.25-3.42)	Ref
Unstratified	22	26.1 (20.5-32.5)	0.55	7	1.88 (1.57-2.26)	0.005
**Study design**						
Prospective	31	23.0 (18.0-28.9)	Ref	8	2.83 (1.46-5.50)	Ref
Retrospective	29	18.7 (12.4-27.1)	0.36	10	2.55 (2.08-3.12)	0.95
Mixed design	3	11.6 (2.0-46.1)	-	2	2.22 (0.64-7.68)	-
**Setting**						
ED	8	19.6 (12.5-29.2)	0.84	2	1.70 (0.55-5.23)	-
General ward	42	17.3 (12.6-23.3)	Ref	12	2.83 (1.68-4.78)	Ref
ICU	13	28.1 (21.3-36.1)	0.12	6	1.93 (1.63-2.29)	0.99

OR – odds ratio, ED – emergency department, ICU – intensive care unit, CI – confidence interval, Ref – reference group

### Prevalence of bacterial co-infection in influenza infection

The included studies were conducted in 20 countries, including 15 World Bank high-income, four middle-income, and one low-income country. Twenty-one studies reported data for patients aged 18 years old and below, 29 reported data for patients aged above 18 years old, and four reported data for patients aged above 65 years old. By study periods, eight studies reported data for the pre-pandemic period, 15 reported data during the 2009 influenza pandemic, and 18 reported data for the post-pandemic period; 22 studies reported data over the pre-, during-, and post-pandemic periods. By health care settings, eight studies reported data in ED, 42 in general wards, and 13 in ICU. For the risk of bias, 31 studies had a low risk of bias in study design, 35 had a low risk of bias in the representativeness of the study population, 55 had a low risk of bias in influenza test methods, 51 had a low risk of bias in testing levels for bacteria, and 46 had a low risk of bias in the methods for bacterial confirmation. (see Table S4-6 in the **Online Supplementary Document** for details of individual studies).

The 63 studies reported on 39 762 influenza cases, 5578 of which had bacterial co-infection. The estimated prevalence of influenza bacterial co-infection ranged from 1.3% (95% confidence interval (CI) = 1.0-1.6) to 75.0% (95% CI = 54.4-88.3) across the studies; we found no significant publication bias (Table S4 and Figure S1A in the **Online Supplementary Document**. The meta-estimate of the prevalence of bacterial co-infection was 20.3% (95% CI = 16.0-25.4) with high statistical heterogeneity between the studies (*I^2^* = 98%, *P* < 0.05).

In subgroup analyses, we found no statistically significant difference in the prevalence of bacterial co-infection between patients above 18 years old (22.5%, 95% CI = 16.5-30.0) and those aged 18 years old or below (23.0%, 95% CI = 13.8-35.7) (*P* > 0.05), and between prospective studies (23.0%, 95% CI = 18.0-28.9) and retrospective studies (18.7%, 95% CI = 12.4-27.1) (*P* > 0.05). Similarly, we found no statistically significant difference across health care settings (*P* > 0.05) or between the pandemic and the post-pandemic period (*P* > 0.05), although the point estimate appeared higher for ICU patients and the post-pandemic period than patients in general wards and the pandemic period. The difference between the pre-pandemic and post-pandemic period (*P* > 0.05) was slightly above the threshold of significance. In sensitivity analyses, the point estimate of the pooled prevalence of bacterial co-infection was lower (17.0%) after excluding the 18 small studies that had less than 50 influenza cases. Results of other sensitivity analyses were similar to the main analysis (**Table 1** and Table S7 in the **Online Supplementary Document**).

### The role of influenza bacterial co-infection in disease severity

The 23 studies with data on the role of influenza bacterial co-infection in disease severity were from 11 countries, including 18 from eight World Bank high-income countries and five from three middle-income countries. Two studies reported data for patients 18 years old and below, eight reported data for patients aged above 18 years, and 13 reported data for patients of all ages. By study periods, only one study reported data for the pre-pandemic period, six for the 2009 influenza pandemic and seven for the post-pandemic period; nine studies reported data over the pre- and post-pandemic periods. By health care settings, 14 studies reported data in general wards and seven in ICU; two studies reported data in ED (Table S5 in the **Online Supplementary Document**). Eleven studies had a low risk of bias in study design; 14 in the representativeness of the study population, 21 in influenza test methods, 22 in testing levels for bacteria, 21 in the methods for bacterial confirmation, and 10 studies in the adjustment for confounding.

**Figure 2:**
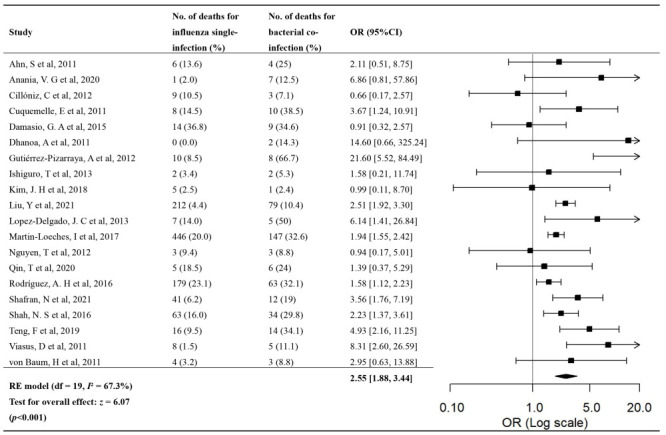
Forest plot of the OR of death for influenza bacterial co-infection compared to influenza single-infection.

Twenty studies provided data on the OR of deaths in patients with bacterial co-infection compared to those with influenza single-infection. The OR of death for bacterial co-infection compared to influenza single-infection ranged from 0.66 (95% CI = 0.17-2.57) to 21.60 (95% CI = 5.52-84.49) across the studies (**Figure 2** and **Table 1**). The OR meta-estimate of death was 2.55 (95% CI = 1.88-3.44) for bacterial co-infection compared to influenza single-infection, with substantial heterogeneity between studies (*I*^2^ = 60.9%, *P* < 0.05). We found no significant publication biases (Figure S1B in the **Online Supplementary Document**).

The OR meta-estimate of death was 2.63 (95% CI = 1.89-3.66) after we excluded the two studies conducted in ED; we found no statistically significant difference in the OR meta-estimate between general wards and ICU, though the point estimate for general wards (OR = 2.83, 95% CI = 1.68-4.78) was higher than for the ICU (OR = 1.93, 95% CI = 1.63-2.29) (*P* > 0.05). By study periods, the pooled OR estimate of death was 2.77 (95% CI = 2.25-3.42) for the post-pandemic period; we found no statistically significant association between influenza bacterial co-infection and mortality for the 2009 influenza pandemic period (OR = 1.91, 95% CI = 0.89-4.11). The pooled OR estimate was 2.11 (95% CI = 1.74-2.56) for people above 18 years old and 3.39 (95% CI = 1.77-6.54) in the studies that provided data for all ages. Only two studies reported data for people 18 years old and below, and the pooled OR estimate of death in the two studies was 2.41 (95% CI = 0.72-8.00). In sensitivity analyses, the pooled OR estimate of death was 2.26 (95% CI = 1.79-2.85) after excluding one influential study. Ten studies had a low risk of bias in the confounding of comorbidities, and the pooled OR estimate of death was 2.08 (95% CI = 1.44-3.00) in these studies (see Table S8-9 in the **Online Supplementary Document** for data on individual studies). Results from other sensitivity analyses were similar to the main analysis (Table S7 in the **Online Supplementary Document**).

Based on the estimates of prevalence of influenza bacterial co-infection and OR of death, we estimated that 23.8% (95% UR = 14.5-35.2) of influenza-associated deaths were attributable to bacterial co-infection. By health care settings, 24.3% (95% UR = 9.8-41.7) of influenza-associated deaths were attributable to bacterial co-infection in general ward, and the estimate was 15.0% (95% UR = 11.7-18.6) in ICU (**Table 2**).

**Table 2:** Estimates of the proportion of influenza deaths that are attributable to bacterial co-infection

	Prevalence of bacterial co-infection, % (95% CI)	Death for bacterial co-infection, OR (95% CI)	Proportion of influenza deaths that are attributable to bacterial co-infection, % (95% UR)
All settings	20.3 (16.0-25.4)	2.55 (1.88-3.44)	23.8 (14.5-35.2)
General ward	17.3 (12.6-23.3)	2.83 (1.68-4.78)	24.3 (9.8-41.7)
ICU	28.1 (21.3-36.1)	1.93 (1.63-2.29)	15.0 (11.7-18.6)

CI – confidence interval, UR – uncertainty ranges

**Figure 3:**
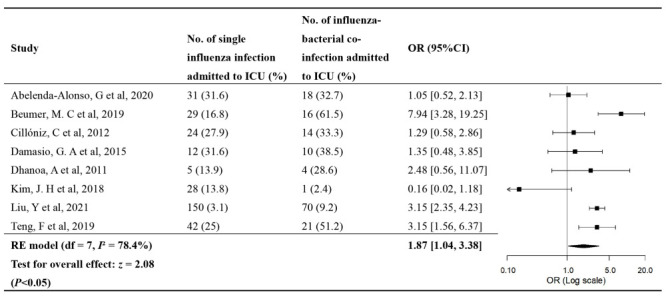
Forest plot of the OR of ICU admission for influenza bacterial co-infection compared to influenza single-infection.

**Figure 4:**
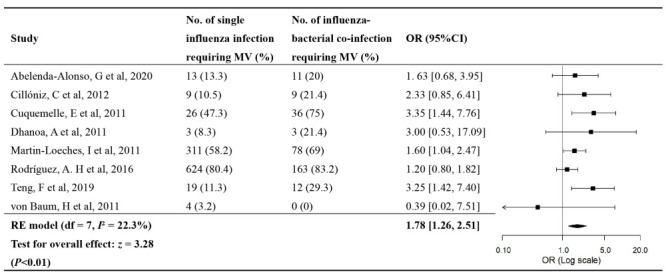
Forest plot of the OR of requirement of MV for influenza bacterial co-infection compared to influenza single-infection.

Eight studies provided data on the OR of ICU admission (**Figure 3**) and requirement of MV (**Figure 4**). No significant publication biases were found (Figure S1 in the **Online Supplementary Document**). The OR meta-estimate of ICU admission was 1.87 (95% CI = 1.04-3.38) for bacterial co-infection compared to influenza single-infection, with substantial heterogeneity between studies (*I*^2^ = 78.4%, *P* < 0.05) (**Figure 3**). Of the eight studies, two were conducted in ED; after excluding them, the pooled OR estimate of ICU admission was 2.35 (95% CI = 1.45- 3.80). The OR meta-estimate of requirement of MV was 1.78 (95% CI = 1.26-2.51) for bacterial co-infection compared to influenza single-infection (*I*^2^ = 22.3%, *P* > 0.05) (**Figure 4**); the OR meta-estimate of requirement of MV was 1.99 (95% CI = 1.47-2.70) after excluding one influential study. Given the small number of studies for these outcomes, no further subgroup analyses were conducted. Details of individual studies are in Table S10-13 in the **Online Supplementary Document**.

Five studies reported the mean or median length of stay in hospitals or ICU for influenza single-infection and bacterial co-infection. In view of the heterogeneity in the outcome (e.g. hospital stay; ICU stay) across studies and the small number of studies, we decided not to perform meta-analysis for this outcome (Table S14-15 in the **Online Supplementary Document**).

## DISCUSSION

To our knowledge, this is the first systematic review to estimate the association of severe outcomes with influenza bacterial co-infection, the proportion of influenza mortality that are attributable to bacterial co-infection. Our results indicate that bacterial co-infection is associated with about 2.6 times higher risk of death than influenza single-infection, and approximately two times higher risk of ICU admission and of requirement of MV than influenza single-infection, and that about one in five influenza deaths are attributable to bacterial co-infection.

Our estimate that 20.3% (95% CI = 16.0-25.4) of influenza infection are co-infected with bacteria is broadly similar to the result (23%, 95% CI = 18-28) of an earlier systematic review [15] including 27 studies published from 1982 to 2014. We included 36 new studies, of which 50% were conducted after the 2009 influenza pandemic. We found that the prevalence of influenza bacterial co-infection was broadly similar by time periods and age groups. Notably, the estimate of the prevalence of influenza bacterial co-infection seemed higher in ICU patients (28%) than hospitalised patients (17%), but with wide and overlapping uncertainty intervals for the two estimates. This was probably related to the substantial uncertainty in estimates of individual studies and the differences between studies.

Our OR estimates highlight that hospitalised patients with influenza bacterial co-infection have approximately two times higher risk of ICU admission and requiring MV, and those admitted to ICU have approximately two times higher risk of death compared to influenza single-infection; the attributable fraction estimates show that nearly one-quarter of influenza-associated deaths could be attributable to bacterial co-infection. These results are in line with previous epidemiological reports. Burk et al [16] conducted a systematic review on clinical significance of viral-bacterial co-infection; based on 31 studies published from 2005 to 2015, they found that viral-bacterial co-infection was associated with increased risk of mortality (OR = 2.1, 95% CI = 1.32-3.31) compared to viral single-infection. Another systematic review on pandemic influenza A(H1N1)pdm09 estimated that bacterial co-infection were diagnosed in 23% of fatal influenza A(H1N1)pdm09 cases [6], but did not estimate the risk of severe outcomes associated with influenza bacterial co-infection or the attributable fraction of influenza bacterial co-infection, focusing rather on pandemic influenza A(H1N1)pdm09 only [3]. Our results are consistent with existing evidence that synergy between influenza viruses and bacteria can lead to dysregulation of host immune responses, augmented inflammatory responses, and immune-mediated host damage; additionally, bacteria express virulence factors that promote viral pathogenesis, leading to increased viral load and decreased clearance. These cause severe complications [17,18]. Although we found no association between influenza bacterial co-infection and death in paediatric and ED patients during the 2009 pandemic or in studies with a mixed study design, data in these subgroups were mostly scarce.

Our study has limitations. Methodological differences were found between studies, which could have affected the estimates. The estimated prevalence of influenza bacterial co-infection remained similar in sensitivity analyses of studies with a low risk of bias in influenza test methods, bacterial confirmation methods, and after excluding potentially influential studies (Table S8 in the **Online Supplementary Document**). The prevalence of bacterial co-infection in prospective studies (23.0%, 95% CI = 18.0-28.9) was broadly similar to retrospective studies (18.7%, 95% CI = 12.4-27.1) (*P* > 0.05). The prevalence of bacterial co-infection could have been underestimated given that not all the influenza cases were tested for bacteria and the reduced bacterial detections associated with many factors (e.g. use of antibiotics before sampling). Fifty-nine studies (94%) tested a broad range of bacterial species and the remaining four tested a few bacterial species (i.e. *Staphylococcus*, *Mycoplasma pneumoniae*, *Legionella pneumophila*, *Streptococcus pneumoniae*); data were not sufficient to allow for analyses by bacterial species, and the estimates could have been affected by the bacterial species that were tested. Few studies reported the adjusted OR estimates of severe outcomes for influenza bacterial co-infection; the unadjusted OR estimate could have been affected by confounding biases. Among others, we consider the presence of co-morbidities an important factor that could have biased the OR estimates of the included studies; the OR estimate of death for bacterial co-infection decreased by 18% among the studies in which the prevalence of co-morbidities was similar between patients with influenza single-infection and those with bacterial co-infection (studies with a low risk of bias in confounding adjustment). Uneven distribution and mixture of confounders across the studies could also introduce biases in the OR estimates. Few studies reported testing levels of bacteria, and differences in testing practice of bacteria in patients with and without severe illnesses could bias the OR estimates. We did not investigate influenza co-infection with other pathogens (e.g. viruses), which might have confounded the relationship of severe outcomes and influenza bacterial co-infection. An earlier systematic review, however, found similar clinical disease severity between viral co-infection and respiratory viral single-infection [11]. This is a systemic review of observational studies and, therefore, causal inference cannot be made, and the association found between bacterial co-infection in patients with influenza and worse clinical outcomes should be interpreted carefully. OR estimates of death in some of the studies were of limited precision, necessitating studies with larger sample sizes. There were limited data on the OR of ICU admission and requirement of MV; the estimates for these outcomes need to be interpreted with caution. Seventy-five percent of the included studies were from World Bank high-income countries; the estimates of prevalence and outcome of influenza bacterial co-infection may be different in low- and middle-income countries, possibility related to heterogeneities in epidemiology of bacterial infection, use of bacterial vaccines (e.g. pneumococcal vaccines), and accessibility to health care resources. More research from low- and middle-income countries is needed to understand how the estimates differ in these countries. Data in this review were from hospitals and ED, and the estimates may not be generalisable to the patients from primary care (e.g. outpatient).

## CONCLUSIONS

Our results highlight the increased risk of ICU admission, requirement of MV and death associated for influenza bacterial co-infection, and about one in four influenza deaths could be attributable to bacterial co-infection. Prevention (e.g. influenza vaccine and pneumococcal vaccine), identification, and effective treatment for suspected bacterial co-infection may reduce the prevalence and severity of influenza bacterial co-infection.

## Additional material


Online Supplementary Document
