# Supplementary material for: The prevalence of influenza bacterial co-infection and its role in disease severity: A systematic review and meta-analysis
Source: J Glob Health. 2023 Jun 16;13:04063. doi: 10.7189/jogh.13.04063 (PMC10270314; doi:10.7189/jogh.13.04063)
Supplement: Online Supplementary Document [file jogh-13-04063-s001.pdf]

Supplementary materials for "The prevalence of influenza bacterial co-infection and its role in disease severity: a systematic review and meta-analysis".

Table S1. Search terms of the study.
Table S2. The test methods and specimen for influenza virus and bacteria.
Table S3. Assessment of risk of biases for studies.
Table S4. Studies reporting data on the prevalence of influenza bacterial co-infection.
Table S5. Characteristics of studies provided data on the severity associated with influenza bacterial co-infection.
Table S6. The risk of bias of the studies reporting data on the prevalence of bacterial co-infection in influenza infections.
Table S7. Results of sensitivity analyses.
Table S8. Studies providing data on the odds ratio of death for the bacterial co-infection.
Table S9. The risk of bias of the studies providing data on the odds ratio of death for the bacterial co-infections.
Table S10. Studies providing data on the odds ratio of ICU admission for the bacterial co-infections.
Table S11. The risk of bias of the studies providing data on the odds ratio of ICU admission for the bacterial co-infection.
Table S12. Studies providing data on the odds ratio of requiring MV for the bacterial co-infection.
Table S13. The risk of bias of the studies providing data on the odds ratio of requirement of mechanical ventilation (MV) for the bacterial co-infection.
Table S14. Studies providing data on the length of hospital stay (LOS).
Table S15. The risk of bias of the studies reporting data on the length of hospital stay (LOS).
Figure S1. Funnel plots of each outcome.

Table S1. Search terms of the study.

Concepts	Web of Science‡	PubMed‡‡	
	Topic words	MeSH	Text words
Influenza	Influenza; flu; influenza virus; H3N2; H1N1; H1N1pdm09	Influenza, Human; Influenza virus; Influenza Vaccines	Flu; H3N2; Grippe; Influenza virus; 2009 H1N1; H1N1; H1N1pdm09; influenza
Bacterial infection	bacteremia; bacterial coinfection; bacterial co-infection; bacterial infection; bacterial pathogen; bacterial pneumonia; bacterial-viral infection; viral-bacterial infection	Bacterial Infections; Pneumonia, Bacterial	bacteremia; bacterial coinfection; bacterial co-infection; bacterial co-infections; bacterial coinfections; bacterial infection; bacterial infections; bacterial pathogen; bacterial pathogens; bacterial pneumonia; bacterial-viral infection; bacterial-viral infections; viral-bacterial infection; viral-bacterial infections; bacterial disease; bacterial diseases; infection, bacterial; infections, bacterial; Bacterial Pneumonia; Bacterial Pneumonias; Pneumonias, Bacterial; Pneumonia, Bacterial
Coinfection	Co infection; Coinfection; Co-infection; concomitant infection; dual infection; polymicrobial infection; mixed infection; secondary infection	Coinfection	Co infection; Co infections; Coinfection; Coinfections; Co-infection; Co-infections; concomitant infection; concomitant infections; dual infection; dual infections; Infection, Polymicrobial; Infections, Polymicrobial; Polymicrobial Infection; Polymicrobial Infections; Infection, Mixed; Infections, Mixed; Mixed Infection; Mixed Infections; Secondary Infection; Secondary Infections; Infection, Secondary; Infections, Secondary

‡: We restrict database to Web of Science core collection, and selected 'TS = ()' to search. We use "OR" to combine the topic words within each concept, such as 'TS = (Influenza, Human OR Influenza virus B)'. We restrict the searches to the timeframe from January 1, 2010 to December 31, 2021.

‡‡: We combine the 'Mesh' and 'Keywords' within each concept using 'OR', e.g., '"influenza, human"[MeSH Terms] OR "Flu"[Text Word]'. We restrict the SPECIES to 'humans' and restrict the searches to the timeframe from January 1, 2010 to December 31, 2021.

Table S2. The test methods and specimen for influenza virus and bacteria.

Study (n = 63)	Test methods for influenza	Specimen for influenza test	Test methods for bacteria	Specimen for bacteria test
Abelenda-Alonso, G et al, 2020[1]	PCR	NPS	cell culture and microscopy; ELISA; rapid immunochromatographic assay, standard serologic methods	blood; sputum; urine
Ahn, S et al, 2011[2]	PCR	NPS	culture; serum enzyme immunoassay; cold agglutinin test; UAT	bronchial aspirates; blood; serum; urine; sputum
Anania, V. G et al, 2020[3]	PCR	NPS; ETA	culture	ETA; blood; pleural fluid
Aston, S. J et al, 2019[4]	PCR	NPS	bacterial culture; UAT; Xpert MTB/RIF assay (Cepheid)	blood; sputum; urine; pleural fluid
Bal, A et al, 2020[5]	PCR	BAL	PCR; bacterial culture; UAT	BAL; urine
Bello, S et al, 2014[6]	PCR	NPS	bacterial culture; Gram staining and culture; sandwich immunoassays and time-resolved amplified cryptate emission (TRACE) measurement	blood; sputum
Bender, J. M et al, 2010[7]	DFA	NPS	Bacterial culture	blood; pleural fluid; cerebrospinal fluid
Beumer, M. C et al, 2019[8]	PCR	nose/throat swabs, sputum; BAL	bacterial culture; UAT	endotracheal or endobronchial secretions; urine
Bjarnason, A et al, 2012[9]	PCR	throat swab	Gram-stain and bacterial culture; UAT	Sputum; blood; urine
Cantais, A et al, 2014[10]	PCR; IFA	NPS	PCR; bacterial culture; pneumococcal antigenuria	blood; aspirate; sputum
Chavan, R. D et al, 2015[11]	PCR	nasal swabs; throat swabs	PCR	clinical samples
Chen, J. Y et al, 2018[12]	PCR	NPS; throat swabs; sputum; BAL	Bacterial culture	NPAs; sputum; blood; BALF
Choi, S. H et al, 2015[13]	PCR	BAL	Bacterial culture	BALF; NPS; sputum; ETA; blood
Cillóniz, C et al, 2012[14]	PCR	NPS	ELISA; immunoenzymatic comercial method; rapid immunochromatographic assay	sputum; blood; urine
Crotty, M. P et al, 2015[15]	respiratory panel assay	NPS	respiratory panel assay; bacterial culture; UAT; direct-fluorescent	urine; NPS

Cuquemelle, E et al, 2011[16]	PCR	NPS; BAL	bacterial culture; UAT; culture of a respiratory tract secretions sample	blood; urine; respiratory tract secretions
D Onofrio, V et al, 2021[17]	PCR	NPS	chest X-ray abnormalities; UAT	urine
Damasio, G. A et al, 2015[18]	PCR	NPS; BAL	Seeplex PneumoBacter ACE Detection kit	NPAs; BAL
Dawood, F. S et al, 2010[19]	NA	NA	cerebrospinal fluid culture	blood; cerebrospinal fluid; pleural fluid; tissue specimen; ETA; sputum
Dhanoa, A et al, 2011[20]	PCR	NA	particle agglutination test; bacterial culture	sputum; tracheal/nasopharyngeal; blood; pleural fluid aspirate; BAL
Esterman, E. E et al, 2013[21]	PCR	NA	DIFT; bacterial culture	pleural fluid; blood
Falsey, A. R et al, 2013[22]	PCR	nose swab ;throat swab	bacterial culture; sputum culture and Gram stain; UAT; pneumococcal serologic testing; PCR	nose swab; throat swab; blood; sputum; urine
Garg, S et al, 2015[23]	PCR; a rapid antigen test; viral culture; DFA; IFA	NA	bacterial culture	blood, pleural and cerebrospinal fluid, BAL, deep tissue biopsy; sputum, ETA
Guo, L. X et al, 2019[24]	PCR	nasal wash products	bacterial culture	blood; sputum
Gutiérrez-Pizarraya, A et al, 2012[25]	PCR	NPS	bacterial cultures; UAT	blood; urine; bronchial aspirates; BAL; sputum
Hagerman, A et al, 2015[26]	PCR	NPS	NA	NA
Hayashi, Y et al, 2012[27]	PCR	NA	bacterial culture; UAT	blood; urine; respiratory tract
Hernández-Bou, S et al, 2013[28]	PCR	NPS	PCR; pneumococcal antigen detection; bacterial culture	sterile fluid; pleural fluid; blood
Ho, Z. J et al, 2015[29]	PCR	Nasal wash samples	PCR	Nasal wash samples
Holter, J. C et al, 2015[30]	PCR	NPS; OPS	bacterial culture; Loeffler stain; Gram stain (optional) and culture; specific antigen detection; PCR	blood; sputum; Urine; BAL; NPS; OPs
Hon, K. L et al, 2010[31]	DIFT	NPAs; BAL; TAs; oral swabs	bacterial culture	sputum; blood
Huijskens, E. G. W et al, 2014[32]	PCR	throat swab; sputum	PCR	throat swab; sputum; urine; serum; blood
Ishiguro, T et al, 2013[33]	Paired sera; RIDT	NPS	UAT; ELISA; bacterial culture	Sputum; Transbronchial aspirate; Bronchial washing; BAL; Transthoracic aspiration; Blood; Pleural fluid

Khandaker, G et al, 2014[34]	PCR; IFA; viral culture; rapid antigen testing	NPAs; nose and throat swab	bacterial culture	blood; sterile site; respiratory specimens
Kim, H. J et al, 2018[35]	PCR	NPS	bacterial culture; respiratory tract specimen cultures; UAT	sputum; blood; urine; ETA; BAL
Kim, H. S et al, 2011[36]	PCR	NA	bacterial culture	sputum; blood; urine; BAL; ascites
Kim, J. H et al, 2018[37]	PCR; IFT	NPS	bacterial culture	blood; sputum; pleural fluid; ETA
Kumar, S et al, 2010[38]	PCR	NPS	bacterial cultures	blood; cerebrospinal fluid; ETA
Lee, E. H et al, 2010[39]	PCR	NPS	PCR; enzyme immunoassay; direct fluorescent antibody	lung and bronchial tissue
Liderot, K et al, 2013[40]	IFT; PCR	Flocked swabs; NPAs	bacterial culture; airway cultures	airways(nasopharynx,throat); blood; sputum; BAL
Lin, C et al, 2019[41]	PCR	sputum	bacterial culture	sputum
Liu, Y et al, 2021[42]	IFA	blood	IFA	blood
Lopez-Delgado, J. C et al, 2013[43]	PCR	nasal flock swab; NPAs; tracheal aspirate; bronchial aspirate; BAL	bacterial culture; Matrix-assisted laser desorption/ionization-time of flight mass spectrometry	sputum; tracheal aspirates; BAL; bronchial washing; pleural biopsy; blood
Lu, Y. Q et al, 2013[44]	PCR	NPS	bacterial culture	blood; respiratory specimens
Malato, L et al, 2011[45]	PCR	Nasalswabs; throat swabs; BAL	bacterial culture	sputum; blood; NPS
Martin-Loeches, I et al, 2011[46]	PCR; Roche detection kit	nasal swabs; BAL; respiratory secretions	bacterial culture; serology	NA
Martin-Loeches, I et al, 2017[47]	PCR	NPS; lower respiratory secretions	ELISA; immunochromatographic test; bacterial culture	Blood; urine; BAL; pleural effusion
Nguyen, T et al, 2012[48]	PCR	NPS	bacterial culture	pleural effusions; ETA; blood; BAL
Nolan, V. G et al, 2018[49]	PCR	sterile site; respiratory secretions; blood	NA	sterile site; respiratory secretions; blood
Poulakou, G et al, 2012[50]	PCR	NPS; OPS	bacterial culture; PCR	blood; pleural fluid; BAL; NPS; OPS
Qin, T et al, 2020[51]	PCR	NA	bacterial culture; UAT	Sputum; blood; urine
Rodríguez, A. H et al, 2016[52]	PCR	NPS	bacterial culture	NPS; blood; alveolar lavage fluid; air intubation; sputum
Shafran, N et al, 2021[53]	PCR	NA	UAT; bacterial culture; Serology tests	blood; pleural fluid; ETA; BAL; urine

Shah, N. S et al, 2016[54]	PCR	NPS; OPS	bacterial culture; GeneXpert assay	blood; sputum; BAL
Shibli, F et al, 2010[55]	PCR; a rapid test; viral culture	NA	bacterial culture	pleural fluid; sputum; tracheal or bronchoscopic sample
Teng, F et al, 2019[56]	PCR	NPS	bacterial culture; UAT; PCR	blood; urine; sputum; NPS
Tief, F et al, 2016[57]	PCR	NA	bacterial cultures; antibodies test	pleural fluid; sputum; serum samples; blood; BAL
Viasus, D et al, 2013[58]	PCR	NPS; OPS;	bacterial culture	Brushings of the posterior pharyngeal wall
Viasus, D et al, 2011[59]	PCR	NPS; BAL	bacterial culture; UAT	blood; normally sterile fluids; sputum; urine
von Baum, H et al, 2011[60]	PCR; viral culture	NA	bacterial culture; UAT	blood; sterile fluids; sputum; urine
Wei, L et al, 2015[61]	PCR	throat washings	bacterial culture; Gram-staining and culture	Urine; blood; sputum; pleural fluid; tracheobronchial aspirates; PBS; BALF
Zhang, Q. L et al, 2011[62]	PCR	NPAs	bacterial culture	NPAs

PCR = polymerase chain reaction. IFA = Indirect immunofluorescence assays. DIFA = direct immunofluorescence assays. RIDT = Rapid influenza diagnostic test. IFT = immunofluorescence testing. NPS = nasopharyngeal swab. ETA = endotracheal aspirate. BAL = bronchoalveolar lavage. OPS = oropharyngeal swab. TAs = tracheal aspirates. NPAs = nasopharyngeal aspirates. ELISA = enzyme linked immunosorbent assay. UAT = urine antigen test. NPAs = nasopharyngeal aspirates. NA = not available.

Table S3. Assessment of risk of biases for studies.

Category	Description	Risk of bias
Study design	Studies where the cases are prospectively enrolled	Low
	Other studies	High
Representativeness of the study population	Good representativeness:<br>≥90% of people with respiratory infections are tested for influenza AND those who are tested positive for influenza are included in this study.	Low
	Including a selected group that may affect the representativeness, e.g.,<br><90% of people with respiratory infections are tested for influenza; OR<br>Not all people who are tested positive for influenza are included in the study; OR<br>Excluding the patients who have high-risk conditions; OR<br>Excluding patients with certain pathogens	High
Influenza test methods	PCR; OR<br>Using other diagnostic tests, but confirming negative samples with PCR	Low
	Other diagnostic tests, e.g., culture, IFA, DFA; OR<br>No mention of diagnostic tests.	High
Bacterial testing level	The prevalence of testing is available AND<br>≥90% of influenza-confirmed cases are tested for bacteria; OR<br>A systematic sample of influenza-confirmed cases are tested for bacteria.	Low
	<90% of influenza-confirmed cases are tested for bacteria, and the cases are not sampled systematically; OR<br>The prevalence of influenza-confirmed cases that are tested for bacteria is unavailable.	High
Bacterial confirmation	High specificity: Culture or nucleic acid detection tests (e.g. PCR) of sputum or lower respiratory tract samples (e.g. ETA, BAL or pleural effusion); OR<br>blood culture; OR<br>antigen detection of urine; AND<br>≥90% of influenza-confirmed cases are tested any above samples.	Low
	Low specificity:<br>Culture or nucleic acid detection tests (e.g. PCR) of upper respiratory tract samples (e.g. oropharyngeal swab, throat swab, nasopharyngeal swab, nasopharyngeal aspirate or nasopharyngeal wash); AND<br><90% of influenza-confirmed cases are tested any samples with high specificity.	High

Category	Description	Risk of bias
Accounting for the adjustment for confounders (only limited to the studies providing data on severity)	Adjusting for potential confounders, e.g. age, the presence of underlying medical conditions; OR<br>No significant differences in the distribution of potential confounders between the exposed and unexposed group.	Low
	Not adjusting for confounders to prevent potential confounding biases AND there are significant differences in the distribution of potential confounders (e.g., age and the presence of underlying medical conditions) between the exposed and unexposed group	High
Assessment of the length of hospital stay (LOS) (only limited to the studies providing data on LOS)	Assessment of LOS is accurate:<br>Respiratory illnesses are community-acquired; OR<br>Hospital-acquired cases are included AND for hospital-acquired cases, the hospital stays that are unrelated to the respiratory illness are excluded.	Low
	Hospital-acquired cases are included AND for hospital-acquired cases, the hospital stays that are unrelated to the respiratory illness are included.	High

PCR = polymerase chain reaction. IFA = indirect immunofluorescence assays. DFA = direct fluorescent antibody. ETA = endotracheal aspirate. BAL = bronchoalveolar lavage.

Table S4. Studies reporting data on the prevalence of influenza bacterial co-infection.

Study (n = 63)	Country	study design	Study period‡	Case definition	Age group of the patients‡‡	Settings	Bacterial pathogens‡‡‡	No. of influenza infections	Prevalence of influenza bacterial co-infections (%)
Abelenda-Alonso, G et al, 2020[1]	Spain	P	unstratified	CAP	>18y	IP	Multiple	153	35.9
Ahn, S et al, 2011[2]	Korea	R	during	CAP	>18y	ED	Multiple	60	26.7
Anania, V. G et al, 2020[3]	USA	P	unstratified	influenza infection	<=18y	ICU	Multiple	105	53.3
Aston, S. J et al, 2019[4]	Malawi	P	post	CAP	>18y	IP	Multiple	31	29.0
Bal, A et al, 2020[5]	France	R	post	ARDS	>18y	ICU	Multiple	45	35.6
Bello, S et al, 2014[6]	Spain	P	unstratified	CAP	>18y	ED	Multiple	40	40.0
Bender, J. M et al, 2010[7]	USA	R	pre	influenza infection	<=18y	IP	Multiple	833	1.9
Beumer, M. C et al, 2019[8]	Netherlands	R	post	ARI	unstratified	IP	Multiple	199	13.1
Bjarnason, A et al, 2012[9]	Iceland	P	unstratified	CAP	>18y	IP	Multiple	22	13.6
Cantais, A et al, 2014[10]	France	P	post	CAP	<=18y	ED	Multiple	16	18.8
Chavan, R. D et al, 2015[11]	India	R	post	ARI	unstratified	IP	Multiple	34	11.8
Chen, J. Y et al, 2018[12]	China	P	unstratified	ARI	<=18y	IP	Multiple	78	15.4
Choi, S. H et al, 2015[13]	Korea	P	unstratified	pneumonia	unstratified	ICU	Multiple	51	37.3
Cillóniz, C et al, 2012[14]	Spain	P	during	pneumonia	>18y	IP	Multiple	128	32.8
Crotty, M. P et al, 2015[15]	USA	P	post	pneumonia	>18y	IP	Multiple	69	14.5
Cuquemelle, E et al, 2011[16]	France	R	during	CAP	unstratified	ICU	Multiple	103	46.6
D Onofrio, V et al, 2021[17]	Belgium	P&R	post	influenza infection	>18y	ED	Multiple	103	10.7
Damasio, G. A et al, 2015[18]	Brazil	R	during	ARI	unstratified	IP	Multiple	64	40.6
Dawood, F. S et al, 2010[19]	USA	P&R	pre	influenza infection	<=18y	IP	Multiple	4015	1.9
Dhanoa, A et al, 2011[20]	Malaysia	R	unstratified	ILI	unstratified	IP	Multiple	50	28.0
Esterman, E. E et al, 2013[21]	Australia	P	during	influenza infection	unstratified	IP	Staphylococcus aureus, Coagulase-negative Staphylococcus	32	18.8
Falsey, A. R et al, 2013[22]	USA	P	unstratified	RTI	>18y	ED	Multiple	90	12.2

Garg, S et al, 2015[23]	USA	R	pre	influenza infection; pneumonia	>18y	IP	Multiple	4765	2.7
Guo, L. X et al, 2019[24]	China	R	post	pneumonia	unstratified	IP	Multiple	205	42.0
Gutiérrez-Pizarraya, A et al, 2012[25]	Spain	P	post	CAP	unstratified	IP	NA	130	9.2
Hagerman, A et al, 2015[26]	Switzerland	R	during	influenza infection	<=18y	IP	Multiple	326	11.0
Hayashi, Y et al, 2012[27]	Australia	R	during	influenza infection	unstratified	IP	Multiple	4491	1.3
Hernández-Bou, S et al, 2013[28]	Spain	R	during	ILI; IBC	<=18y	ED	Multiple	308	9.4
Ho, Z. J et al, 2015[29]	Singapore	P	unstratified	FRI	>18y	IP	Multiple	1372	13.4
Holter, J. C et al, 2015[30]	Norway	P	unstratified	CAP	>18y	IP	Multiple	40	57.5
Hon, K. L et al, 2010[31]	China	R	unstratified	influenza infection	<=18y	ICU	Multiple	18	33.3
Huijskens, E. G. W et al, 2014[32]	Netherlands	P	pre	CAP	>18y	ED	Multiple	32	46.9
Ishiguro, T et al, 2013[33]	Japan	R	unstratified	CAP	unstratified	IP	Multiple	97	42.3
Khandaker, G et al, 2014[34]	Australia	P	during	CAP	<=18y	IP	Multiple	506	6.5
Kim, H. J et al, 2018[35]	Korea	R	unstratified	Pneumonia	>18y	ICU	Multiple	21	57.1
Kim, H. S et al, 2011[36]	Korea	R	during	ILI	unstratified	IP	Multiple	115	33.9
Kim, J. H et al, 2018[37]	Korea	R	unstratified	Pneumonia	unstratified	ED	Mycoplasma pneumoniae	244	16.8
Kumar, S et al, 2010[38]	USA	R	during	influenza infection	unstratified	IP	Multiple	75	1.3
Lee, E. H et al, 2010[39]	USA	R	during	influenza infection	unstratified	IP	Multiple	47	27.7
Liderot, K et al, 2013[40]	Sweden	R	pre	IBC	unstratified	IP	Multiple	1094	6.5
Lin, C et al, 2019[41]	China	R	post	CAP	unstratified	IP	Multiple	21	23.8
Liu, J et al, 2020[63]	China	R	post	RTI	<=18y	IP	Multiple	1720	16.2
Liu, Y et al, 2021[42]	China	R	post	RTI	>18y	IP	Multiple	5575	13.7
Lopez-Delgado, J. C et al, 2013[43]	Spain	P	unstratified	CAP	>18y	ICU	Multiple	60	16.7
Lu, Y. Q et al, 2013[44]	China	P	unstratified	RTI	<=18y	IP	Multiple	18	55.6
Malato, L et al, 2011[45]	France	R	during	ARDS	unstratified	ICU	Multiple	24	20.8
Martin-Loeches, I et al, 2011[46]	Spain	P	unstratified	CARC	unstratified	ICU	Multiple	645	17.5
Martin-Loeches, I et al, 2017[47]	Spain	P	unstratified	CARC	>18y	ICU	Multiple	2901	16.6
Nguyen, T et al, 2012[48]	USA	P&R	during	CARC	<=18y	IP	Multiple	66	51.5
Nolan, V. G et al, 2018[49]	USA	P	unstratified	CAP	<=18y	IP	Multiple	149	18.1
Poulakou, G et al, 2012[50]	Spain	P	unstratified	ARI	unstratified	ICU	Multiple	53	15.1

Qin, T et al, 2020[51]	China	P	post	IBC	unstratified	IP	Multiple	52	48.1
Rodríguez, A. H et al, 2016[52]	Spain	P	unstratified	CARC	>18y	ICU	Multiple	972	20.2
Shafran, N et al, 2021[53]	Israel	R	post	IBC	unstratified	IP	Multiple	724	8.7
Shah, N. S et al, 2016[54]	USA	R	post	IBC	unstratified	ICU	Multiple	507	25.4
Shibli, F et al, 2010[55]	Israel	R	pre	CAP	>18y	IP	Multiple	24	75.0
Teng, F et al, 2019[56]	China	P	post	CAP	unstratified	IP	Multiple	209	19.6
Tief, F et al, 2016[57]	Germany	R	post	ILI	<=18y	IP	Multiple	411	48.2
Viasus, D et al, 2013[58]	Spain	P	post	CAP	>18y	IP	Multiple	115	12.2
Viasus, D et al, 2011[59]	Spain	P	during	influenza infection	unstratified	IP	Multiple	585	7.7
von Baum, H et al, 2011[60]	Germany	P	pre	pneumonia	unstratified	IP	Multiple	160	21.3
Wei, L et al, 2015[61]	China	P	unstratified	ARI; pneumonia	<=18y	IP	Multiple	551	22.0
Zhang, Q. L et al, 2011[62]	China	P	pre	CAP	<=18y	IP	Multiple	75	20.0

P = prospective. R = retrospective. P&R = prospective and retrospective. AFRS = acute febrile respiratory syndrome. ARDS = acute respiratory distress syndrome. ARI = acute respiratory infection. CAP = community-acquired pneumonia. CARC = community-acquired respiratory coinfection. FRI = febrile respiratory illnesses. IBC = influenza bacterial coinfection. ILI = influenza-like illness. RTI = respiratory tract infection. ED = emergency department. IP = general ward. ICU = intensive care unit. NA = unavailable.

‡: If study time overlapped two or three time periods, we unstratified the study. pre = pre-pandemic 2009. during = during pandemic 2009. post = post-pandemic 2009. unstratified = time periods crossover in the study time.

‡‡: If the age of included patients overlapped children and adults, the study would not be stratified.

‡‡‡: If types of involved bacteria more than two, we defined the study as multiple pathogens (MP). Multiple = multiple pathogens. NA = unavailable.

Table S5. Characteristics of studies provided data on the severity associated with influenza bacterial co-infection.

Study (n = 23)	Country	No. of patients	study design	Study period‡	Case definition	Age group of the patients‡‡	Co-morbidities	Reported outcomes			
								Death	ICU	MV	LOS
Abelenda-Alonso, G et al, 2020[1]	Spain	153	P	unstratified	CAP	>18y	CH	No	Yes	Yes	No
Ahn, S et al, 2011[2]	Korea	60	R	during	CAP	>18y	CS	Yes	No	No	No
Anania, V. G et al, 2020[3]	USA	105	P	unstratified	influenza infection	<=18y	CH	Yes	No	No	Yes
Beumer, M. C et al, 2019[8]	Netherlands	199	R	post	ARI	unstratified	NA	No	Yes	No	No
Cillóniz, C et al, 2012[14]	Spain	128	P	during	pneumonia	>18y	CS	Yes	Yes	Yes	Yes
Cuquemelle, E et al, 2011[16]	France	103	R	during	CAP	unstratified	SH	Yes	No	Yes	Yes
Damasio, G. A et al, 2015[18]	Brazil	64	R	during	ARI	unstratified	SH	Yes	Yes	No	Yes
Dhanoa, A et al, 2011[20]	Malaysia	50	R	unstratified	ILI	unstratified	CH	Yes	Yes	Yes	No
Gutiérrez-Pizarraya, A et al, 2012[25]	Spain	130	P	post	CAP	unstratified	NA	Yes	No	No	No
Ishiguro, T et al, 2013[33]	Japan	97	R	unstratified	CAP	unstratified	NA	Yes	No	No	No
Kim, J. H et al, 2018[37]	Korea	244	R	unstratified	Pneumonia	unstratified	SH	Yes	Yes	No	Yes
Liu, Y et al, 2021[42]	China	5575	R	post	RTI	>18y	NA	Yes	Yes	No	No
Lopez-Delgado, J. C et al, 2013[43]	Spain	60	P	unstratified	CAP	>18y	NA	Yes	No	No	No
Martin-Loeches, I et al, 2011[46]	Spain	647	P	unstratified	CARC	unstratified	CS	No	No	Yes	No

Martin-Loeches, I et al, 2017[47]	Spain	2684	P	unstratified	CARC	>18y	NA	Yes	No	No	No
Nguyen, T et al, 2012[48]	USA	66	P&R	during	CARC	<=18y	NA	Yes	No	No	No
Qin, T et al, 2020[51]	China	52	P	post	IBC	unstratified	NA	Yes	No	No	No
Rodríguez, A. H et al, 2016[52]	Spain	972	P	unstratified	CARC	>18y	CS	Yes	No	Yes	No
Shafran, N et al, 2021[53]	Israel	724	R	post	IBC	unstratified	NA	Yes	No	No	No
Shah, N. S et al, 2016[54]	USA	507	R	post	IBC	unstratified	CS	Yes	No	No	No
Teng, F et al, 2019[56]	China	209	R	post	CAP	unstratified	CS	Yes	Yes	Yes	No
Viasus, D et al, 2011[59]	Spain	585	P	during	influenza infection	unstratified	NA	Yes	No	No	No
von Baum, H et al, 2011[60]	Germany	160	P	pre	pneumonia	>18y	NA	Yes	No	Yes	No
n								20	8	8	5

P = prospective. R = retrospective. P&R = prospective and retrospective. AFRS = acute febrile respiratory syndrome. ARDS = acute respiratory distress syndrome. ARI = acute respiratory infection. CAP = community-acquired pneumonia. CARC = community-acquired respiratory coinfection. FRI = febrile respiratory illnesses. IBC = influenza bacterial coinfection. ILI = influenza-like illness. RTI = respiratory tract infection. CH = influenza bacterial co-infection group has statistically higher comorbidity rate; SH = influenza single infection group has statistically higher comorbidity rate; CS = comorbidity rate is statistical similar between two groups. NA = the information about comorbidity rate is unavailable

‡: If study time overlapped two or three time periods, we unstratified the study. pre = pre-pandemic 2009. during = during pandemic 2009. post = post-pandemic 2009. unstratified = time periods crossover in the study time.

‡‡: If the age of included patients overlapped the two age groups, the study would not be stratified.

Table S6. The risk of bias of the studies reporting data on the prevalence of bacterial co-infection in influenza infections.

Study (n = 63)	Study design	Representativeness of population	Test methods for influenza	Testing levels for bacteria	Methods for bacterial confirmation
Abelenda-Alonso, G et al, 2020[1]	Low	High	Low	Low	Low
Ahn, S et al, 2011[2]	High	Low	Low	Low	Low
Anania, V. G et al, 2020[3]	Low	High	Low	Low	Low
Aston, S. J et al, 2019[4]	Low	High	Low	Low	Low
Bal, A et al, 2020[5]	High	High	Low	Low	Low
Bello, S et al, 2014[6]	Low	High	Low	Low	Low
Bender, J. M et al, 2010[7]	High	High	High	Low	Low
Beumer, M. C et al, 2019[8]	High	Low	Low	Low	Low
Bjarnason, A et al, 2012[9]	Low	High	Low	Low	Low
Cantais, A et al, 2014[10]	Low	Low	Low	Low	Low
Chavan, R. D et al, 2015[11]	High	Low	Low	High	High
Chen, J. Y et al, 2018[12]	Low	Low	Low	High	Low
Choi, S. H et al, 2015[13]	Low	High	Low	Low	High
Cillóniz, C et al, 2012[14]	Low	High	Low	Low	Low
Crotty, M. P et al, 2015[15]	Low	Low	High	High	High
Cuquemelle, E et al, 2011[16]	High	Low	Low	Low	Low
D Onofrio, V et al, 2021[17]	High	High	Low	High	High
Damasio, G. A et al, 2015[18]	High	High	Low	Low	High
Dawood, F. S et al, 2010[19]	High	Low	High	Low	Low
Dhanoa, A et al, 2011[20]	High	Low	Low	Low	Low
Esterman, E. E et al, 2013[21]	Low	Low	Low	Low	Low
Falsey, A. R et al, 2013[22]	Low	High	Low	Low	Low
Garg, S et al, 2015[23]	High	Low	Low	Low	Low
Guo, L. X et al, 2019[24]	High	Low	Low	Low	Low
Gutiérrez-Pizarraya, A et al, 2012[25]	Low	Low	Low	High	Low
Hagerman, A et al, 2015[26]	High	Low	Low	High	High
Hayashi, Y et al, 2012[27]	High	High	Low	Low	High
Hernández-Bou, S et al, 2013[28]	High	Low	Low	Low	Low
Ho, Z. J et al, 2015[29]	Low	High	Low	Low	High

Holter, J. C et al, 2015[30]	Low	High	Low	Low	Low
Hon, K. L et al, 2010[31]	High	High	High	Low	Low
Huijskens, E. G. W et al, 2014[32]	Low	High	Low	Low	High
Ishiguro, T et al, 2013[33]	High	High	High	Low	Low
Khandaker, G et al, 2014[34]	Low	Low	Low	High	Low
Kim, H. J et al, 2018[35]	High	Low	Low	High	Low
Kim, H. S et al, 2011[36]	High	Low	Low	High	Low
Kim, J. H et al, 2018[37]	High	High	Low	Low	Low
Kumar, S et al, 2010[38]	High	High	Low	Low	Low
Lee, E. H et al, 2010[39]	High	High	Low	High	Low
Liderot, K et al, 2013[40]	High	Low	Low	High	High
Lin, C et al, 2019[41]	High	High	Low	Low	Low
Liu, Y et al, 2021[42]	High	High	Low	Low	Low
Lopez-Delgado, J. C et al, 2013[43]	Low	Low	Low	Low	Low
Lu, Y. Q et al, 2013[44]	Low	Low	Low	Low	High
Malato, L et al, 2011[45]	High	Low	Low	Low	High
Martin-Loeches, I et al, 2011[46]	Low	Low	Low	Low	Low
Martin-Loeches, I et al, 2017[47]	Low	Low	Low	Low	Low
Nguyen, T et al, 2012[48]	High	High	High	Low	Low
Nolan, V. G et al, 2018[49]	Low	High	Low	Low	High
Poulakou, G et al, 2012[50]	Low	Low	Low	High	Low
Qin, T et al, 2020[51]	Low	Low	Low	Low	High
Rodríguez, A. H et al, 2016[52]	Low	Low	Low	Low	Low
Shafran, N et al, 2021[53]	High	Low	Low	Low	Low
Shah, N. S et al, 2016[54]	High	Low	Low	Low	Low
Shibli, F et al, 2010[55]	Low	High	Low	Low	High
Teng, F et al, 2019[56]	High	Low	Low	Low	Low
Tief, F et al, 2016[57]	High	Low	Low	Low	High
Viasus, D et al, 2013[58]	Low	Low	Low	Low	Low
Viasus, D et al, 2011[59]	Low	Low	Low	Low	Low
von Baum, H et al, 2011[60]	Low	High	Low	Low	Low
Wei, L et al, 2015[61]	Low	Low	Low	Low	High

Zhang, Q. L et al, 2011[62]	Low	High	High	Low	Low

Table S7. Results of sensitivity analyses.

Sensitivity analyses	Prevalence of bacterial co-infection (n = 63)		OR of death for bacterial co-infection (n = 20)	
	n‡	Prevalence (%) (95% CI)	n‡	OR (95% CI)
influenza test methods	56	20.3 (16.2, 25.0)	18	2.67 (1.93, 3.69)
bacterial confirmation	46	19.7 (15.1, 25.4)	18	2.72 (1.97, 3.75)
whether the study accounted for potential confounders	-	-	10	2.08 (1.44, 3.00)
excluding influential studies	62	19.7 (15.6, 24.6)	19	2.26 (1.79, 2.85)
excluding small studies	46	17.0 (12.7, 22.4)	-	-

We conducted sensitivity analyses on the two main outcomes: prevalence of bacterial co-infection in influenza patients, the OR of death for influenza bacterial co-infection compared to influenza single infection. The terms of sensitivity analyses were predefined.

‡: The number of studies represented those studies with low bias of each term.

Table S8. Studies providing data on the odds ratio of death for the bacterial co-infection.

Study (n = 20)	Country	Study period‡	Case definition	Age group of the patients‡‡	Settings	Co-morbidities	Influenza single-infection		Bacterial co-infection		Odds ratio (95%CI)
							No. of patients	No. of deaths	No. of patients	No. of deaths	
Ahn, S et al, 2011[2]	Korea	during	CAP	>18y	ED	CS	44	6	16	4	2.11 [0.51, 8.75]
Anania, V. G et al, 2020[3]	USA	unstratified	influenza infection	<=18y	ICU	CH	49	1	56	7	6.86 [0.81, 57.86]
Cillóniz, C et al, 2012[14]	Spain	during	CAP	>18y	IP	CS	86	9	42	3	0.66 [0.17, 2.57]
Cuquemelle, E et al, 2011[16]	France	during	CAP	unstratified	ICU	SH	55	8	26	10	3.67 [1.24, 10.91]
Damasio, G. A et al, 2015[18]	Brazil	during	ARI	unstratified	IP	SH	38	14	26	9	0.91 [0.32, 2.57]
Dhanoa, A et al, 2011[20]	Malaysia	unstratified	ILI	unstratified	IP	CH	36	0	14	2	14.60 [0.66, 325.24]
Gutiérrez-Pizarraya, A et al, 2012[25]	Spain	post	CAP	unstratified	IP	NA	118	10	12	8	21.60 [5.52, 84.49]
Ishiguro, T et al, 2013[33]	Japan	unstratified	CAP	unstratified	IP	NA	59	2	38	2	1.58 [0.21, 11.74]
Kim, J. H et al, 2018[37]	Korea	unstratified	pneumonia	unstratified	ED	SH	203	5	41	1	0.99 [0.11, 8.70]
Liu, Y et al, 2021[42]	China	post	RTI	>18y	IP	NA	4814	212	761	79	2.51 [1.92, 3.30]
Lopez-Delgado, J. C et al, 2013[43]	Spain	unstratified	CAP	>18y	ICU	NA	50	7	10	5	6.14 [1.41, 26.84]
Martin-Loeches, I et al, 2017[47]	Spain	unstratified	CAC	>18y	ICU	NA	2233	446	451	147	1.94 [1.55, 2.42]
Nguyen, T et al, 2012[48]	USA	during	CAC	<=18y	IP	NA	32	3	34	3	0.94 [0.17, 5.01]
Qin, T et al, 2020[51]	China	post	secondary bacterial infection	unstratified	IP	NA	27	5	25	6	1.39 [0.37, 5.29]

Rodríguez, A. H et al, 2016[52]	Spain	unstratified	CAC	>18y	ICU	CS	776	179	196	63	1.58 [1.12, 2.23]
Shafran, N et al, 2021[53]	Israel	post	bacterial coinfections and secondary infection	unstratified	IP	NA	661	41	63	12	3.56 [1.76, 7.19]
Shah, N. S et al, 2016[54]	USA	post	Bacterial coinfection	>18y	ICU	CS	393	63	114	34	2.23 [1.37, 3.61]
Teng, F et al, 2019[56]	China	post	CAP	>18y	IP	CS	168	16	41	14	4.93 [2.16, 11.25]
Viasus, D et al, 2011[59]	Spain	during	influenza infection	unstratified	IP	NA	540	8	45	5	8.31 [2.60, 26.59]
von Baum, H et al, 2011[60]	Germany	pre	pneumonia	>18y	IP	NA	126	4	34	3	2.95 [0.63, 13.88]

AFRS = acute febrile respiratory syndrome. ARDS = acute respiratory distress syndrome. ARI = acute respiratory infection. CAP = community-acquired pneumonia. CARC = community-acquired respiratory coinfection. RI = febrile respiratory illnesses. IBC = influenza bacterial coinfection. ILI = influenza-like illness. RTI = respiratory tract infection. ED = emergency department. IP = general ward. ICU = intensive care unit. CH = influenza bacterial co-infection group has statistically higher comorbidity rate; SH = influenza single infection group has statistically higher comorbidity rate; CS = comorbidity rate is statistical similar between two groups. NA = the information about comorbidity rate is unavailable.

‡: If study time overlapped two or three time periods, we unstratified the study. pre = pre-pandemic 2009. during = during pandemic 2009. post = post-pandemic 2009. unstratified = time periods crossover in the study time.

‡‡: If the age of included patients overlapped the two age groups, the study would not be stratified.

Table S9. The risk of bias of the studies providing data on the odds ratio of death for the bacterial co-infections.

Study (n = 20)	Study design	Representativeness of population	Test methods for influenza	Testing levels for bacteria	Methods for bacterial confirmation	Confounding adjustment
Ahn, S et al, 2011[2]	High	Low	Low	Low	Low	Low
Anania, V. G et al, 2020[3]	Low	High	Low	Low	Low	Low
Cillóniz, C et al, 2012[14]	Low	High	Low	Low	Low	Low
Cuquemelle, E et al, 2011[16]	High	Low	Low	Low	Low	Low
Damasio, G. A et al, 2015[18]	High	High	Low	Low	High	Low
Dhanoa, A et al, 2011[20]	High	Low	Low	Low	Low	Low
Gutiérrez-Pizarraya, A et al, 2012[25]	Low	Low	Low	High	Low	High
Ishiguro, T et al, 2013[33]	High	High	High	Low	Low	High
Kim, J. H et al, 2018[37]	High	High	Low	Low	Low	Low
Liu, Y et al, 2021[42]	High	High	Low	Low	Low	High
Lopez-Delgado, J. C et al, 2013[43]	Low	Low	Low	Low	Low	High
Martin-Loeches, I et al, 2017[47]	Low	Low	Low	Low	Low	High
Nguyen, T et al, 2012[48]	High	High	High	Low	Low	High
Qin, T et al, 2020[51]	Low	Low	Low	Low	High	High
Rodríguez, A. H et al, 2016[52]	Low	Low	Low	Low	Low	Low
Shafran, N et al, 2021[53]	High	Low	Low	Low	Low	High
Shah, N. S et al, 2016[54]	High	Low	Low	Low	Low	Low
Teng, F et al, 2019[56]	High	Low	Low	Low	Low	Low
Viasus, D et al, 2011[59]	Low	Low	Low	Low	Low	High
von Baum, H et al, 2011[60]	Low	High	Low	Low	Low	High

Table S10. Studies providing data on the odds ratio of ICU admission for the bacterial co-infections.

Study (n = 8)	Country	Study period‡	Case definition	Age group of the patients‡‡	Settings	Influenza single-infection		Bacterial co-infection		Odds ratio (95%CI)
						The number of patients	The number of patients admitted to ICU	The number of patients	The number of patients admitted to ICU	
Abelenda-Alonso, G et al, 2020[1]	Spain	unstratified	CAP	>18y	IP	98	31	55	18	1.05 [0.52, 2.13]
Beumer, M. C et al, 2019[8]	Netherlands	post	ARI	unstratified	IP	173	29	26	16	7.94 [3.28, 19.25]
Cillóniz, C et al, 2012[14]	Korea	during	CAP	>18y	IP	86	24	42	14	1.29 [0.58, 2.86]
Damasio, G. A et al, 2015[18]	Brazil	during	ARI	unstratified	IP	38	12	26	10	1.35 [0.48, 3.85]
Dhanoa, A et al, 2011[20]	Malaysia	unstratified	ILI	unstratified	IP	36	5	14	4	2.48 [0.56, 11.07]
Kim, J. H et al, 2018[37]	Korea	unstratified	pneumonia	unstratified	ED	203	28	41	1	0.16 [0.02, 1.18]
Liu, Y et al, 2021[42]	China	post	RTI	>18y	IP	4814	150	761	70	3.15 [2.35, 4.23]
Teng, F et al, 2019[56]	China	post	CAP	unstratified	IP	168	42	41	21	3.15 [1.56, 6.37]

CAP = community-acquired pneumonia. ARI = acute respiratory infection. ILI = influenza-like illness. RTI = respiratory tract infection. ED = emergency department. IP = general ward.

‡: If study time overlapped two or three time periods, we unstratified the study. during = during pandemic 2009. post = post-pandemic 2009. unstratified = time periods crossover in the study time.

‡‡: If the age of included patients overlapped the two age groups, the study would not be stratified.

Table S11. The risk of bias of the studies providing data on the odds ratio of ICU admission for the bacterial co-infection.

Study (n = 8)	Study design	Representativeness of population	Test methods for influenza	Testing levels for bacteria	Methods for bacterial confirmation	Accounting for confounding
Abelenda-Alonso, G et al, 2020[1]	Low	High	Low	Low	Low	Low
Beumer, M. C et al, 2019[8]	High	Low	Low	Low	Low	High
Cillóniz, C et al, 2012[14]	Low	High	Low	Low	Low	Low
Damasio, G. A et al, 2015[18]	High	High	Low	Low	High	Low
Dhanoa, A et al, 2011[20]	High	Low	Low	Low	Low	Low
Kim, J. H et al, 2018[37]	High	High	Low	Low	Low	Low
Liu, Y et al, 2021[42]	High	High	Low	Low	Low	Low
Teng, F et al, 2019[56]	High	High	Low	Low	Low	High

Table S12. Studies providing data on the odds ratio of requiring MV for the bacterial co-infection.

Study (n = 8)	Country	Study period‡	Case definition	Age group of the patients‡‡	Settings	Influenza single-infection		Bacterial co-infection		Odds ratio (95%CI)
						The number of patients	The number of patients requiring MV	The number of patients	The number of patients requiring MV	
Abelenda-Alonso, G et al, 2020[1]	Spain	unstratified	CAP	>18y	IP	98	13	55	11	1.63 [0.68, 3.95]
Cillóniz, C et al, 2012[14]	Spain	during	CAP	>18y	IP	86	9	42	9	2.33 [0.85, 6.41]
Cuquemelle, E et al, 2011[16]	France	during	CAP	unstratified	ICU	55	26	48	36	3.35 [1.44, 7.76]
Dhanoa, A et al, 2011[20]	Malaysia	unstratified	ILI	unstratified	IP	36	3	14	3	3.00 [0.53, 17.09]
Martin-Loeches, I et al, 2011[46]	Spain	unstratified	CARC	unstratified	ICU	534	311	113	78	1.60 [1.04, 2.47]
Rodríguez, A. H et al, 2016[52]	Spain	unstratified	CARC	>18y	ICU	776	624	196	163	1.20 [0.80, 1.82]
Teng, F et al, 2019[56]	China	post	CAP	unstratified	IP	168	19	41	12	3.25 [1.42, 7.40]
von Baum, H et al, 2011[60]	Germany	pre	pneumonia	>18y	IP	126	4	34	0	0.39 [0.02, 7.51]

CAP = community-acquired pneumonia. ILI = influenza-like illness. CARC = community-acquired respiratory coinfection. IP = general ward. ICU = intensive care unit.

‡: If study time overlapped two or three time periods, we unstratified the study. pre = pre-pandemic 2009. during = during pandemic 2009. post = post-pandemic 2009. unstratified = time periods crossover in the study time.

‡‡: If the age of included patients overlapped the two age groups, the study would not be stratified.

Table S13. The risk of bias of the studies providing data on the odds ratio of requirement of mechanical ventilation (MV) for the bacterial co-infection.

Study (n = 8)	Study design	Representativeness of population	Test methods for influenza	Testing levels for bacteria	Methods for bacterial confirmation	Accounting for confounding
Abelenda-Alonso, G et al, 2020[1]	Low	High	Low	Low	Low	Low
Cillóniz, C et al, 2012[14]	Low	High	Low	Low	Low	Low
Cuquemelle, E et al, 2011[16]	High	Low	Low	Low	Low	Low
Dhanoa, A et al, 2011[20]	High	Low	Low	Low	Low	Low
Martin-Loeches, I et al, 2011[46]	Low	Low	Low	Low	Low	High
Rodríguez, A. H et al, 2016[52]	Low	Low	Low	Low	Low	Low
Teng, F et al, 2019[56]	High	Low	Low	Low	Low	Low
von Baum, H et al, 2011[60]	Low	High	Low	Low	Low	High

Table S14. Studies providing data on the length of hospital stay (LOS).

Study (n = 5)	Country	Study period‡	Case definition	Age group of the patients‡‡	Settings	The mean LOS of the influenza single-infections (SD)	The mean LOS of the bacterial co-infections (SD)	mean difference between the groups (95%CI)
Anania, V. G et al, 2020[3]	USA	unstratified	influenza infection	<=18y	ICU	7.5 (5.1)	11.0 (10.0)	3.5 | 0.5, 6.5]
Cillóniz, C et al, 2012[14]	Spain	during	CAP	>18y	IP	5.7 (4.5)	6.7 (3.8)	1.0 [-0.5, 2.5]
Cuquemelle, E et al, 2011[16]	France	during	CAP	unstratified	ICU	8.0 (10.0)	15.3 (19.4)	7.3 [-1.2, 15.9]
Damasio, G. A et al, 2015[18]	Brazil	during	ARI	unstratified	IP	6.5 (7.3)	4.2 (3.5)	-2.3 |-5.0, 0.4]
Kim, J. H et al, 2018[37]	Korea	unstratified	pneumonia	unstratified	ED	6.7 (6.0)	5.0 (3.1)	-1.7 [-2.9, -0.4]

CAP = community-acquired pneumonia. ILI = influenza-like illness. CARC = community-acquired respiratory coinfection. ED = emergency department. IP = general ward. ICU = intensive care unit.

‡: If study time overlapped two or three time periods, we unstratified the study. during = during pandemic 2009. unstratified = time periods crossover in the study time.

‡‡: If the age of included patients overlapped the two age groups, the study would not be stratified.

Table S15. The risk of bias of the studies reporting data on the length of hospital stay (LOS).

Study (n = 5)	Study design	Representativeness of population	Test methods for influenza	Testing levels for bacteria	Methods for bacterial confirmation	Accounting for confounders	Accuracy of hospital stay (LOS)
Anania, V. G et al, 2020[3]	Low	High	Low	Low	Low	Low	High
Cillóniz, C et al, 2012[14]	Low	High	Low	Low	Low	Low	Low
Cuquemelle, E et al, 2011[16]	High	Low	Low	Low	Low	High	Low
Damasio, G. A et al, 2015[18]	High	High	Low	Low	High	High	Low
Kim, J. H et al, 2018[37]	High	High	Low	Low	Low	High	Low

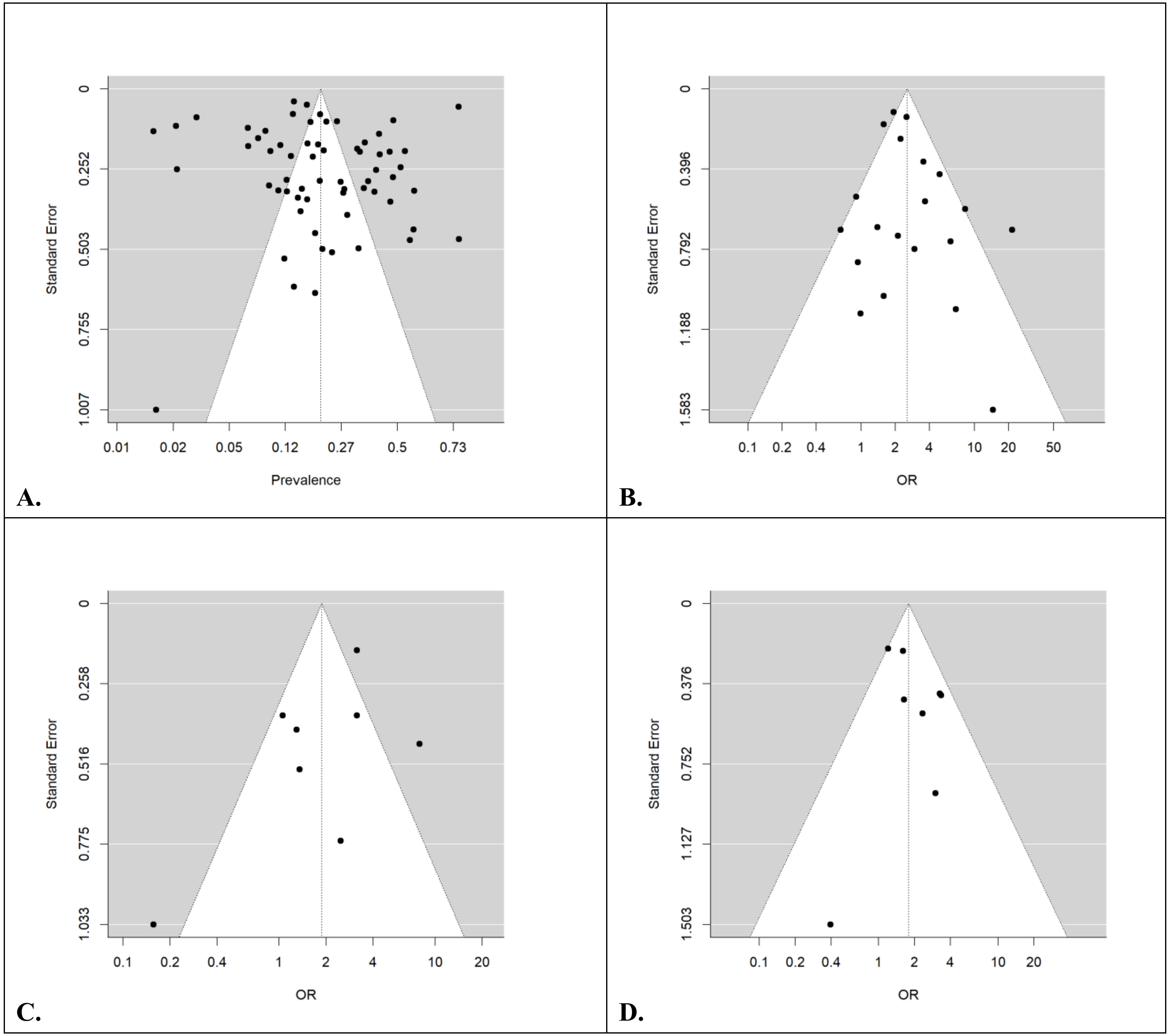

Figure S1. Funnel plots of each outcome. Panel A. The funnel plot presents the publication bias of 63 articles reporting eligible data on the prevalence of bacterial co-infection in influenza infection. It appears symmetric and Egger's test of asymmetry was insignificant for bias (Kendall's tau = 0.10, *P* = 0.27). **Panel B.** The funnel plot presents the publication bias of 20 articles reporting eligible data on the OR of deaths

in patients with bacterial co-infection compared to those with influenza single-infection. It appears symmetric and Egger's test of asymmetry was insignificant for bias (Kendall's tau = 0.07, *P* = 0.68). **Panel C.** The funnel plot presents the publication bias of eight articles reporting eligible data on the OR of ICU admission in patients with bacterial co-infection compared to those with influenza single-infection. It appears symmetric and Egger's test of asymmetry was insignificant for bias (Kendall's tau = -0.29, *P* = 0.40). **Panel D.** The funnel plot presents the publication bias of eight articles reporting eligible data on the OR of requirement of MV in patients with bacterial co-infection compared to those with influenza single-infection. It appears symmetric and Egger's test of asymmetry was insignificant for bias (Kendall's tau = 0.14, *P* = 0.72).

Reference

[1] Abelenda-Alonso G, Rombauts A, Gudiol C, Meije Y, Ortega L, Clemente M, et al. Influenza and Bacterial Coinfection in Adults With Community-Acquired Pneumonia Admitted to Conventional Wards: Risk Factors, Clinical Features, and Outcomes. Open Forum Infectious Diseases. 2020;7(3).

[2] Ahn S, Kim WY, Kim SH, Hong S, Lim CM, Koh Y, et al. Role of procalcitonin and C-reactive protein in differentiation of mixed bacterial infection from 2009 H1N1 viral pneumonia. Influenza and other respiratory viruses. 2011;5(6):398-403.

[3] Anania VG, Randolph AG, Yang XY, Nguyen A, Newhams MM, Mathews WR, et al. Early Amplified Respiratory Bioactive Lipid Response Is Associated With Worse Outcomes in Pediatric Influenza-Related Respiratory Failure. Open Forum Infectious Diseases. 2020;7(5).

[4] Aston SJ, Ho A, Jary H, Huwa J, Mitchell T, Ibitoye S, et al. Etiology and Risk Factors for Mortality in an Adult Community-acquired Pneumonia Cohort in Malawi. American journal of respiratory and critical care medicine. 2019;200(3):359-69.

[5] Bal A, Casalegno JS, Melenotte C, Daviet F, Ninove L, Edouard S, et al. Influenza-induced acute respiratory distress syndrome during the 2010-2016 seasons: bacterial co-infections and outcomes by virus type and subtype. Clinical microbiology and infection : the official publication of the European Society of Clinical Microbiology and Infectious Diseases. 2020;26(7):947.e1-.e4.

[6] Bello S, Minchole E, Fandos S, Lasierra AB, Ruiz MA, Simon AL, et al. Inflammatory response in mixed viral-bacterial community-acquired pneumonia. BMC pulmonary medicine. 2014;14.

[7] Bender JM, Ampofo K, Gesteland P, Sheng XM, Korgenski K, Raines B, et al. Influenza Virus Infection in Infants Less Than Three Months of Age. Pediatric Infectious Disease Journal. 2010;29(1):6-9.

[8] Beumer MC, Koch RM, van Beuningen D, OudeLashof AM, van de Veerdonk FL, Kolwijck E, et al. Influenza virus and factors that are associated with ICU admission, pulmonary co-infections and ICU mortality. Journal of Critical Care. 2019;50:59-65.

[9] Bjarnason A, Thorleifsdottir G, Löve A, Gudnason JF, Asgeirsson H, Hallgrimsson KL, et al. Severity of influenza A 2009 (H1N1) pneumonia is underestimated by routine prediction rules. Results from a prospective, population-based study. PloS one. 2012;7(10):e46816.

[10] Cantais A, Mory O, Pillet S, Verhoeven PO, Bonneau J, Patural H, et al. Epidemiology and microbiological investigations of community-acquired pneumonia in children admitted at the emergency department of a university hospital. Journal of clinical virology : the official publication of the Pan American Society for Clinical Virology. 2014;60(4):402-7.

[11] Chavan RD, Kothari ST, Zunjarrao K, Chowdhary AS. Surveillance of acute respiratory infections in Mumbai during 2011-12. Indian journal of medical microbiology. 2015;33(1):43-50.

[12] Chen JY, Hu PW, Zhou T, Zheng TL, Zhou LX, Jiang CP, et al. Epidemiology and clinical characteristics of acute respiratory tract infections among hospitalized infants and young children in Chengdu, West China, 2009-2014. Bmc Pediatrics. 2018;18.

[13] Choi SH, Huh JW, Hong SB, Lee JY, Kim SH, Sung H, et al. Clinical characteristics and outcomes of severe rhinovirus-associated pneumonia identified by bronchoscopic bronchoalveolar lavage in adults: comparison with severe influenza virus-associated pneumonia. Journal of clinical virology : the official publication of the Pan American Society for Clinical Virology. 2015;62:41-7.

[14] Cillóniz C, Ewig S, Menéndez R, Ferrer M, Polverino E, Reyes S, et al. Bacterial co-infection with H1N1 infection in patients admitted with community acquired pneumonia. The Journal of infection. 2012;65(3):223-30.

[15] Crotty MP, Meyers S, Hampton N, Bledsoe S, Ritchie DJ, Buller RS, et al. Epidemiology, Co-Infections, and Outcomes of Viral Pneumonia in Adults An Observational Cohort Study. Medicine. 2015;94(50).

[16] Cuquemelle E, Soulis F, Villers D, Roche-Campo F, Ara Somohano C, Fartoukh M, et al. Can procalcitonin help identify associated bacterial infection in patients with severe influenza pneumonia? A multicentre study. Intensive care medicine. 2011;37(5):796-800.

[17] D'Onofrio V, Van Steenkiste E, Meersman A, Waumans L, Cartuyvels R, Van Halem K, et al. Differentiating

influenza from COVID-19 in patients presenting with suspected sepsis. European journal of clinical microbiology & infectious diseases : official publication of the European Society of Clinical Microbiology. 2021;40(5):987-95.
[18] Damasio GA, Pereira LA, Moreira SD, Duarte dos Santos CN, Dalla-Costa LM, Raboni SM. Does virus-bacteria coinfection increase the clinical severity of acute respiratory infection? Journal of medical virology. 2015;87(9):1456-61.
[19] Dawood FS, Fiore A, Kamimoto L, Bramley A, Reingold A, Gershman K, et al. Burden of seasonal influenza hospitalization in children, United States, 2003 to 2008. The Journal of pediatrics. 2010;157(5):808-14.
[20] Dhanoa A, Fang NC, Hassan SS, Kaniappan P, Rajasekaram G. Epidemiology and clinical characteristics of hospitalized patients with pandemic influenza A (H1N1) 2009 infections: the effects of bacterial coinfection. Virology journal. 2011;8:501.
[21] Esterman EE, Lahra MM, Zurynski YA, Booy R, Elliott EJ. Influenza infection in infants aged < 6 months during the H1N1-09 pandemic: A hospital-based case series. Journal of Paediatrics and Child Health. 2013;49(8):635-40.
[22] Falsey AR, Becker KL, Swinburne AJ, Nylen ES, Formica MA, Hennessey PA, et al. Bacterial Complications of Respiratory Tract Viral Illness: A Comprehensive Evaluation. Journal of Infectious Diseases. 2013;208(3):432-41.
[23] Garg S, Jain S, Dawood FS, Jhung M, Pérez A, D'Mello T, et al. Pneumonia among adults hospitalized with laboratory-confirmed seasonal influenza virus infection-United States, 2005-2008. BMC infectious diseases. 2015;15:369.
[24] Guo LX, Wei D, Zhang XX, Wu YR, Li QY, Zhou M, et al. Clinical Features Predicting Mortality Risk in Patients With Viral Pneumonia: The MuLBSTA Score. Frontiers in Microbiology. 2019;10.
[25] Gutiérrez-Pizarraya A, Pérez-Romero P, Alvarez R, Aydillo TA, Osorio-Gómez G, Milara-Ibáñez C, et al. Unexpected severity of cases of influenza B infection in patients that required hospitalization during the first postpandemic wave. The Journal of infection. 2012;65(5):423-30.
[26] Hagerman A, Posfay-Barbe KM, Duppenthaler A, Heininger U, Berger C, Grp PIS. Clinical characteristics and outcomes in children hospitalised with pandemic influenza A/H1N1/09 virus infection - a nationwide survey by the Pediatric Infectious Diseases Group of Switzerland. Swiss medical weekly. 2015;145.
[27] Hayashi Y, Vaska VL, Baba H, Nimmo GR, Davis L, Paterson DL. Influenza-associated bacterial pathogens in patients with 2009 influenza A (H1N1) infection: impact of community-associated methicillin-resistant Staphylococcus aureus in Queensland, Australia. Internal Medicine Journal. 2012;42(7):755-60.
[28] Hernández-Bou S, Novell CB, Alins JG, García-García JJ. Hospitalized children with influenza A H1N1 (2009) infection: a Spanish multicenter study. Pediatric emergency care. 2013;29(1):49-52.
[29] Ho ZJ, Zhao X, Cook AR, Loh JP, Ng SH, Tan BH, et al. Clinical differences between respiratory viral and bacterial mono- and dual pathogen detected among Singapore military servicemen with febrile respiratory illness. Influenza and other respiratory viruses. 2015;9(4):200-8.
[30] Holter JC, Müller F, Bjørang O, Samdal HH, Marthinsen JB, Jenum PA, et al. Etiology of community-acquired pneumonia and diagnostic yields of microbiological methods: a 3-year prospective study in Norway. BMC infectious diseases. 2015;15:64.
[31] Hon KL, Leung TF, Cheung KL, Ng PC, Chan PK. Influenza and parainfluenza associated pediatric ICU morbidity. Indian journal of pediatrics. 2010;77(10):1097-101.
[32] Huijskens EGW, Koopmans M, Palmen FMH, van Erkel AJM, Mulder PGH, Rossen JWA. The value of signs and symptoms in differentiating between bacterial, viral and mixed aetiology in patients with community-acquired pneumonia. Journal of medical microbiology. 2014;63(Pt 3):441-52.
[33] Ishiguro T, Takayanagi N, Yamaguchi S, Yamakawa H, Nakamoto K, Takaku Y, et al. Etiology and factors contributing to the severity and mortality of community-acquired pneumonia. Internal medicine (Tokyo, Japan). 2013;52(3):317-24.
[34] Khandaker G, Zurynski Y, Ridley G, Buttery J, Marshall H, Richmond PC, et al. Clinical epidemiology and predictors of outcome in children hospitalised with influenza A(H1N1)pdm09 in 2009: a prospective national study. Influenza and other respiratory viruses. 2014;8(6):636-45.
[35] Kim HJ, Choi SM, Lee J, Park YS, Lee CH, Yim JJ, et al. Respiratory virus of severe pneumonia in South Korea: Prevalence and clinical implications. PloS one. 2018;13(6):e0198902.

[36] Kim HS, Kim JH, Shin SY, Kang YA, Lee HG, Kim JS, et al. Fatal cases of 2009 pandemic influenza A (H1N1) in Korea. Journal of Korean medical science. 2011;26(1):22-7.
[37] Kim JH, Kwon JH, Lee JY, Lee JS, Ryu JM, Kim SH, et al. Clinical features of Mycoplasma pneumoniae coinfection and need for its testing in influenza pneumonia patients. Journal of Thoracic Disease. 2018;10(11):6118-+.
[38] Kumar S, Havens PL, Chusid MJ, Willoughby RE, Jr., Simpson P, Henrickson KJ. Clinical and epidemiologic characteristics of children hospitalized with 2009 pandemic H1N1 influenza A infection. The Pediatric infectious disease journal. 2010;29(7):591-4.
[39] Lee EH, Wu C, Lee EU, Stoute A, Hanson H, Cook HA, et al. Fatalities associated with the 2009 H1N1 influenza A virus in New York city. Clinical infectious diseases : an official publication of the Infectious Diseases Society of America. 2010;50(11):1498-504.
[40] Liderot K, Ahl M, Ozenci V. Secondary bacterial infections in patients with seasonal influenza A and pandemic H1N1. BioMed research international. 2013;2013:376219.
[41] Lin C, Chen H, He P, Li Y, Ke C, Jiao X. Etiology and characteristics of community-acquired pneumonia in an influenza epidemic period. Comparative immunology, microbiology and infectious diseases. 2019;64:153-8.
[42] Liu Y, Ling L, Wong SH, Wang MH, Fitzgerald JR, Zou X, et al. Outcomes of respiratory viral-bacterial co-infection in adult hospitalized patients. EClinicalMedicine. 2021;37:100955.
[43] Lopez-Delgado JC, Rovira A, Esteve F, Rico N, Mañez Mendiluce R, Ballús Noguera J, et al. Thrombocytopenia as a mortality risk factor in acute respiratory failure in H1N1 influenza. Swiss medical weekly. 2013;143:w13788.
[44] Lu YQ, Wang SF, Zhang LH, Xu C, Bian CR, Wang ZX, et al. Epidemiology of Human Respiratory Viruses in Children with Acute Respiratory Tract Infections in Jinan, China. Clinical & Developmental Immunology. 2013.
[45] Malato L, Llavador V, Marmier E, Youssef J, Weber CB, Roze H, et al. Pandemic influenza A(H1N1) 2009: molecular characterisation and duration of viral shedding in intensive care patients in Bordeaux, south-west France, May 2009 to January 2010. Eurosurveillance. 2011;16(4):11-7.
[46] Martin-Loeches I, Sanchez-Corral A, Diaz E, Granada RM, Zaragoza R, Villavicencio C, et al. Community-Acquired Respiratory Coinfection in Critically III Patients With Pandemic 2009 Influenza A(H1N1) Virus. Chest. 2011;139(3):555-62.
[47] Martin-Loeches I, Schultz MJ, Vincent JL, Alvarez-Lerma F, Bos LD, Sole-Violan J, et al. Increased incidence of co-infection in critically ill patients with influenza. Intensive care medicine. 2017;43(1):48-58.
[48] Nguyen T, Kyle UG, Jaimon N, Tcharmtchi MH, Coss-Bu JA, Lam F, et al. Coinfection with Staphylococcus aureus increases risk of severe coagulopathy in critically ill children with influenza A (H1N1) virus infection. Critical care medicine. 2012;40(12):3246-50.
[49] Nolan VG, Arnold SR, Bramley AM, Ampofo K, Williams DJ, Grijalva CG, et al. Etiology and Impact of Coinfections in Children Hospitalized With Community-Acquired Pneumonia. Journal of Infectious Diseases. 2018;218(2):179-88.
[50] Poulakou G, Souto J, Balcells J, Pérez M, Laborda C, Roca O, et al. First influenza season after the 2009 pandemic influenza: characteristics of intensive care unit admissions in adults and children in Vall d'Hebron Hospital. Clinical microbiology and infection : the official publication of the European Society of Clinical Microbiology and Infectious Diseases. 2012;18(4):374-80.
[51] Qin T, Geng T, Zhou H, Han Y, Ren H, Qiu Z, et al. Super-dominant pathobiontic bacteria in the nasopharyngeal microbiota as causative agents of secondary bacterial infection in influenza patients. Emerging microbes & infections. 2020;9(1):605-15.
[52] Rodríguez AH, Avilés-Jurado FX, Díaz E, Schuetz P, Trefler SI, Solé-Violán J, et al. Procalcitonin (PCT) levels for ruling-out bacterial coinfection in ICU patients with influenza: A CHAID decision-tree analysis. The Journal of infection. 2016;72(2):143-51.
[53] Shafran N, Shafran I, Ben-Zvi H, Sofer S, Sheena L, Krause I, et al. Secondary bacterial infection in COVID-19 patients is a stronger predictor for death compared to influenza patients. Scientific reports. 2021;11(1):12703.
[54] Shah NS, Greenberg JA, McNulty MC, Gregg KS, Riddell Jt, Mangino JE, et al. Bacterial and viral co-infections complicating severe influenza: Incidence and impact among 507 U.S. patients, 2013-14. Journal of clinical virology : the official publication of the Pan American Society for Clinical Virology. 2016;80:12-9.

[55] Shibli F, Chazan B, Nitzan O, Flatau E, Edelstein H, Blondheim O, et al. Etiology of community-acquired pneumonia in hospitalized patients in northern Israel. The Israel Medical Association journal : IMAJ. 2010;12(8):477-82.
[56] Teng F, Liu X, Guo SB, Li Z, Ji WQ, Zhang F, et al. Community-acquired bacterial co-infection predicts severity and mortality in influenza-associated pneumonia admitted patients. Journal of infection and chemotherapy : official journal of the Japan Society of Chemotherapy. 2019;25(2):129-36.
[57] Tief F, Hoppe C, Seeber L, Obermeier P, Chen X, Karsch K, et al. An inception cohort study assessing the role of pneumococcal and other bacterial pathogens in children with influenza and ILI and a clinical decision model for stringent antibiotic use. Antiviral therapy. 2016;21(5):413-24.
[58] Viasus D, Marinescu C, Villoslada A, Cordero E, Galvez-Acebal J, Farinas MC, et al. Community-acquired pneumonia during the first post-pandemic influenza season: A prospective, multicentre cohort study. Journal of Infection. 2013;67(3):185-93.
[59] Viasus D, Paño-Pardo JR, Pachón J, Campins A, López-Medrano F, Villoslada A, et al. Factors associated with severe disease in hospitalized adults with pandemic (H1N1) 2009 in Spain. Clinical microbiology and infection : the official publication of the European Society of Clinical Microbiology and Infectious Diseases. 2011;17(5):738-46.
[60] von Baum H, Schweiger B, Welte T, Marre R, Suttorp N, Pletz MWR, et al. How deadly is seasonal influenza-associated pneumonia? The German Competence Network for Community-Acquired Pneumonia. European Respiratory Journal. 2011;37(5):1151-7.
[61] Wei L, Liu W, Zhang XA, Liu EM, Wo Y, Cowling BJ, et al. Detection of viral and bacterial pathogens in hospitalized children with acute respiratory illnesses, Chongqing, 2009-2013. Medicine. 2015;94(16):e742.
[62] Zhang QL, Guo ZQ, MacDonald NE. Vaccine Preventable Community-acquired Pneumonia in Hospitalized Children in Northwest China. Pediatric Infectious Disease Journal. 2011;30(1):7-10.
[63] Liu J, Wang M, Zhao Z, Lin X, Zhang P, Yue Q, et al. Viral and bacterial coinfection among hospitalized children with respiratory tract infections. American journal of infection control. 2020;48(10):1231-6.
